# Comparison of two different intraosseous access methods in a physician-staffed helicopter emergency medical service – a quality assurance study

**DOI:** 10.1186/s13049-019-0594-6

**Published:** 2019-02-13

**Authors:** Renate Sørgjerd, Geir Arne Sunde, Jon-Kenneth Heltne

**Affiliations:** 1 Department of Anesthesia and Intensive Care, Haukeland University Hospital, Jonas Lies vei 65, 5021 Bergen, Norway; 2 Department of Clinical Medicine, University of Bergen, Bergen, Norway

**Keywords:** Cardiac arrest, Emergency medical services, Infusions, Intraosseous, EZ-IO, FAST-R, Trauma

## Abstract

**Background:**

Intravenous access in critically ill and injured patients can be difficult or impossible in the field. Intraosseous access is a well-established alternative to achieve access to a noncollapsible vascular network. We wanted to compare the use of a sternal and tibial/humeral intraosseous device in a physician-staffed helicopter emergency medical service.

**Methods:**

The helicopter emergency medical service in Bergen, Norway, is equipped with two different intraosseous devices, the EZ-IO and FAST-Responder. We compared insertion time, insertion sites, flow, indication for intraosseous access, and complications between the tibial/humeral and sternal techniques.

**Results:**

In 49 patients, 53 intraosseous insertions were made. The overall intraosseous rate was 1.5% (53 insertions in 3600 patients treated). The main patient categories were cardiac arrest and trauma. Overall, 93.9% of the insertions were successful on the first attempt. The median insertion time using EZ-IO was 15 s compared to 20 s using FAST-Responder. Insertion complications registered using the EZ-IO included extravasation, aspiration failure and insertion time > 30 s. Using FAST-Responder, there were reported complications such as user failure (12.5%) and insertion time > 30 s (12.5%). Regarding the flow, we found that 35.1% of the EZ-IO insertions experienced poor flow and needed a pressure bag. With FAST-Responder, the flow was reported as very good or good in 85.7%, and no insertions had poor flow.

**Conclusion:**

Intraosseous access seems to be a reliable rescue technique in our helicopter emergency medical service, with high insertion success rates. EZ-IO was a more rapid method in gaining vascular access compared to FAST-Responder. However, FAST-Responder may be a better method when high-flow infusion is needed. Few complications were registered with both techniques in our service.

## Background

The intraosseous (IO) method has been described as a simple and reliable method to achieve vascular access in both cadaver and clinical studies [1, 2]. Studies have shown that IO can be a fast method with few complications, and the method gives access to a noncollapsible vascular network in the intraosseous space [3–5]. However, IO may be contraindicated if the patient has an infection at the site of insertion, traumatic limb injuries or osseous pathology [6, 7]. The IO route is a rapid, simple and safe procedure in both pediatric and adult patients, with an effectiveness equivalent to peripheral venous cannulation in terms of pharmacokinetic and clinical efficacy [1, 4]. In theory, any medication and virtually all types of fluids that can be given intravenously can be infused via an IO access. It has been suggested that hypertonic or strongly alkaline agents should be avoided or diluted as they have been associated with increased incidence of complications [5, 8, 9]. However, we have found that all resuscitation drugs can be delivered through a verified IO access [1, 7].

In our physician-staffed helicopter emergency medical service (HEMS), IO is mainly used as a rescue technique when primary attempts at establishing intravenous access fail [3]. We have previously compared several IO methods (Bone Injection Gun, EZ-IO and Inter V manual bone marrow aspiration needle), and we found that EZ-IO was the most successful method [3]. However, a sternal route was not available during the previous study period. Today, the HEMS is equipped with two types of intraosseous devices: EZ-IO and FAST-Responder (FAST-R). The EZ-IO, a battery powered device, is most commonly used in the proximal and distal tibia, as well as in the humeral head. The FAST-R is a semiautomatic device intended for sternal insertion only. In pediatric patients, the proximal tibia is a well-established site for IO insertion; however, the optimal insertion site for adults is often debated [2]. Regarding IO in pediatric patients, EZ-IO seems to be the better option and is a reliable method in prehospital conditions in all ages [3, 8, 10]. Currently, there are no available FAST-R options for pediatric patients.

Only a few studies have compared sternal to tibial/humeral IO in critically ill patients in a prehospital setting [11, 12]. There is still limited research on sternal IO access, and many studies have been on cadaver models [11]. It has been shown that the FAST-1, the predecessor to FAST-R, has high flow rates [11]. Our objective was to evaluate the use of EZ-IO and FAST-R regarding insertion time and sites, flow, indication for IO and complications in prehospital emergencies.

## Methods

### Study aim

Our primary objectives were to evaluate the insertion times, insertion sites, complications and flow rates in two different intraosseous devices used by our HEMS. The secondary objectives were to describe IO insertion criteria and main patient categories.

### Study design and setting

The study was designed as a quality assurance study in our HEMS based at Haukeland University Hospital, Bergen, Norway. This service is operational 24/7 and is a physician-staffed service. Seven prehospital anesthesiologists and 10 flight paramedics performed the IO insertions and collected the data prospectively. All patients requiring IO from January 1st, 2014 to November 30th, 2016 were included in the study. There were no exclusion criteria.

### Materials

Our HEMS is equipped with two different IO devices: EZ-IO (® Vidacare Corp, San Antonio, Texas) and FAST-Responder (® Pyng Medical Corporation, Vancouver, BC, Canada). EZ-IO is a battery powered device enabling 500–1000 insertions [13]. EZ-IO has three different needle sizes (the smallest is certified down to 3 kg) [14]. It is most commonly used in three different insertion sites (proximal and distal tibia and the humeral head). In 1997, FAST-1 was the first FDA approved sternal IO system. In 2013, PYNG launched FAST-R, a modified version of FAST-1, to meet the needs of civilian emergency medical services and hospital critical care personnel. FAST-R is a disposable semiautomatic device for sternal insertion only, in which the insertion site is located just below the sternal notch. It cannot be used in children < 12 years of age [15]. Only one attempt can be made per FAST- unit, whereas multiple attempts are possible using the EZ-IO [12].

### Data

The data was analyzed for insertion time, flow, indication for IO, insertion site and complications. IO device placement was confirmed by loss of resistance, aspiration of bone marrow or blood, and/or administration of saline and fluid administration. Insertion time was recorded in seconds from placement against skin until vascular access was confirmed by aspiration of bone marrow or infusion of fluids. Flow was evaluated based on drip chamber flow and categorized as very good (continuous flow without pressure bag), good (fast drip without pressure bag), adequate (slow drip without pressure bag) or poor (poor flow and in need of pressure bag). All primary and final insertion sites were recorded. The physicians recorded insertion related complications such as technical issues, extravasation, IO needle detachment, failed aspiration, fracture when inserting, insertion time exceeding 30 s and difficulty in localizing insertion site. Patient journals were checked for all patients admitted to the hospital for late complications, such as osteomyelitis, fracture after IO insertion or compartment syndrome. In case of missing data, the attending prehospital physician was contacted to provide supplemental data.

### Statistics

SPSS statistics (software version 23.0,® SPSS Inc., Chicago, USA) was used for statistical calculations. The Mann-Whitney U-test was conducted to compare insertion time between EZ-IO and FAST-R. A difference in insertion time was considered significant if the *p*-value was < 0.05.

## Results

**Fig. 1:**
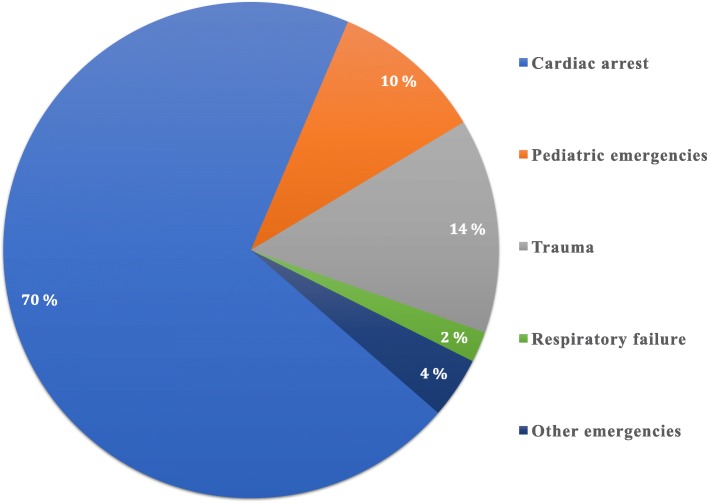
The percentage of the patient categories receiving IO insertion

During the study period, 53 insertions were made in 49 patients, and 69.3% of patients were male. This group included 10.2% pediatric patients < 18 years. The total IO rate was 1.5% (53 insertions in 3600 patients treated). Main patient categories are mentioned in Fig. 1.

### Insertion success

**Fig. 2:**
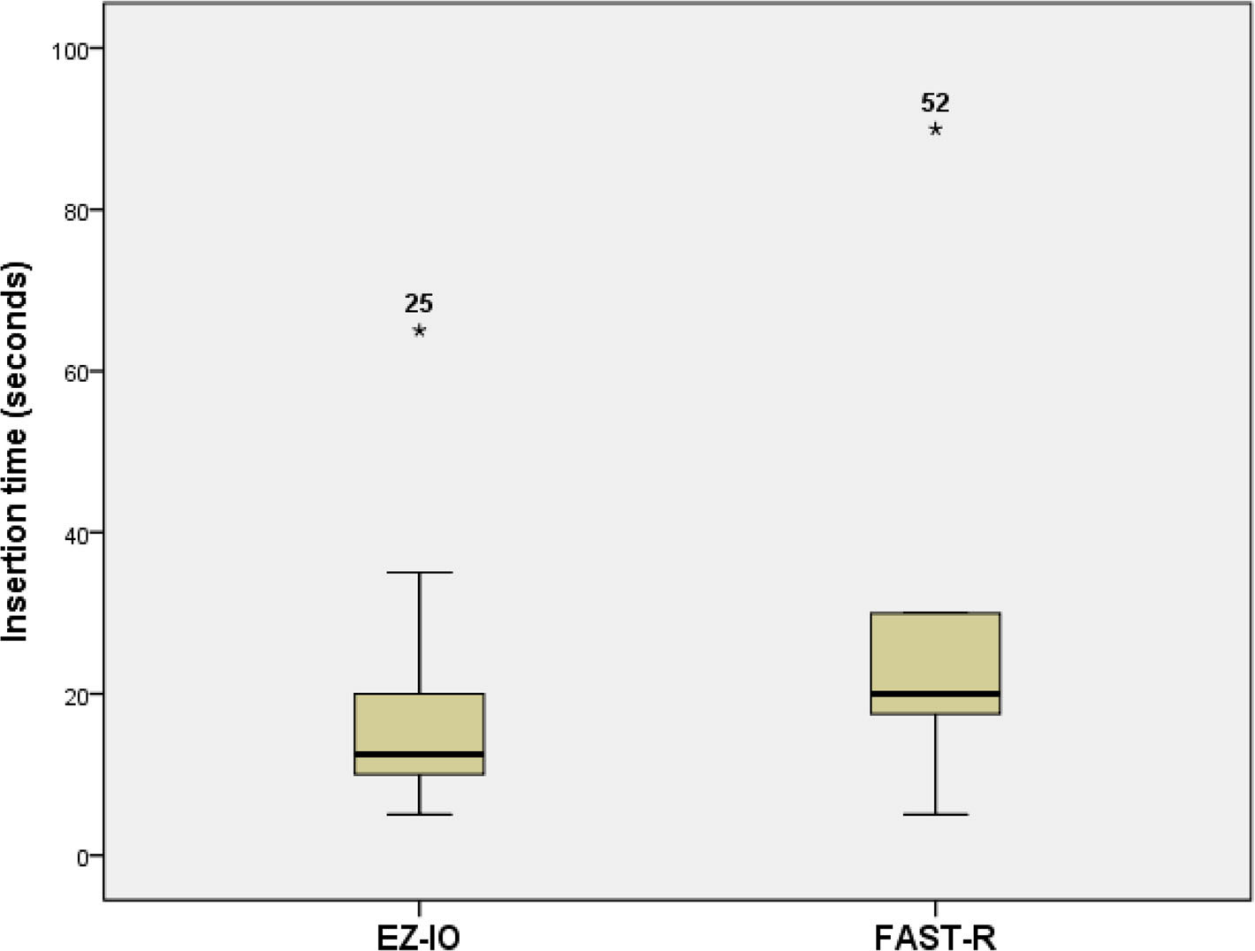
Intraosseous insertion times for EZ-IO and FAST-R, including the identified outliers

**Fig. 3:**
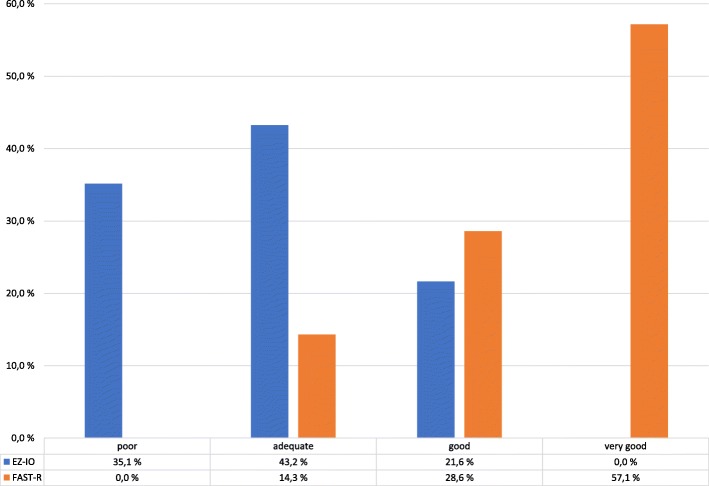
Flow was evaluated based on drip chamber flow and categorized as very good (continuous flow without pressure bag), good (fast drip without pressure bag), adequate (slow drip without pressure bag) or poor (poor flow and in need of pressure bag)

A total of 93.9% of the insertions were successful on the first attempt. The remaining 6.1% of the insertions were successful on the second attempt. The median insertion time using the EZ-IO was shorter compared to FAST-R: 15 s and 20 s, respectively. A significant difference in insertion times was found when we included the outliers (Fig. 2). A comparison of flow is illustrated in Fig. 3.

### Complications

Insertion complications when using EZ-IO included extravasation (2.4%), aspiration failure (11.9%) and insertion time > 30 s (4.8%). Using FAST-R, complications were reported such as user failure (12.5%) and insertion time > 30s (12.5%). It was reported that 8% of the patients experienced pain during infusion. A total of 34.7% of the patients survived until hospital admittance, and 20.4% were alive after 30 days. No cases of long-term insertion complications were described in the in-hospital journals at discharge (or death).

### Insertion criteria

IO was used as a bridge to later IV in 32%. In 28%, the IO insertion was used because of failed IV access. In 15%, patients received IO parallel to IV attempts. All IO insertions were made on critically ill or injured patients.

### Insertion sites

The main EZ-IO insertion site was in the proximal tibia (90.5%); however, 9.5% of the EZ-IO were inserted in the humerus. All FAST-R insertions were sternal (100%). With EZ-IO, 2.4% of the tibial insertions failed, and another successful attempt was made in the humeral head. Of the patients receiving FAST-R, 25% of the insertions were reported as failures. These patients subsequently received successful tibial insertions using the EZ-IO.

### IO administrations

In most cases, the patient needed medications (77.6%) and/or fluids (55.1%). Plasma or whole-blood was administered in 10.2% of cases. In 6.1% of the patients, nothing was administered through the IO.

## Discussion

### Main results

To our knowledge, this is the first study comparing FAST-R and EZ-IO. In general, we experienced a low complication rate using both IO techniques. Our study showed a high insertion success rate on the first attempt, and all insertions were successful after two attempts. Although the EZ-IO may be a faster technique, our study suggests that the FAST-R may have a place when high-flow infusion rates in patients are required.

Calkins et al. described two failures in 31 attempts with sternal placement using FAST-1 caused by lack of continuous increasing pressure over the sternal insertion site [12]. Regarding the FAST-R insertion failures in our study, the patients subsequently received successful IO insertions with the EZ-IO. This finding may indicate that some training with the devices is recommended.

Using the EZ-IO, extravasation occurred in 2.3% of the patients, and aspiration failure was experienced in 11.9% of the cases. In 2.3% of the patients receiving EZ-IO insertions, low battery caused an insertion time > 30s. Compared to FAST-R, this result shows that EZ-IO has an expected shelf-life, which is estimated by the manufacturer to be approximately 10 years or 500 insertions. However, aspiration failure was regarded as a complication and occurred using both IO methods. This issue could result in physicians attempting another IO insertion, even though the previous IO might have been correctly inserted in the intramedullary space [13, 16]. Hammer et al. also showed that both methods are easy to use, even by medical students without any specific training [11]. In a prehospital environment, the physician tends to choose the method he or she is familiar with since IO is mainly used as a rescue technique. Our HEMS has good experience using the EZ-IO, which could explain the low number of FAST-R insertions.

We found a significant difference in insertion times, as we could not exclude the outliers from the data. However, given our low number of FAST-R insertions, the outliers have a huge impact on the analysis. In a study conducted by Hammer et al., no significant difference in insertion times or in first pass insertion success rates were found between EZ-IO and FAST-R [11]. This result might indicate that a higher number of FAST-R insertions could have improved our insertion times since we observed a minimal difference in median insertion times. Nonetheless, comparing studies may be difficult due to different patient populations and study protocols [17].

We found that none of the FAST-R infusions needed a pressure bag to maintain a very good flow. None of the EZ-IO insertions experienced very good flow, and 33.3% needed a pressure bag. Pasley et al. concluded that the sternal IO provided a more consistent and higher flow rates compared to tibial or humeral insertions [2].

Our results also showed a larger number of EZ-IO insertions in the proximal tibia compared to the humerus, which was also the case in a previous study conducted in our HEMS. This site may be preferred in different HEMSs in Norway and other European countries due to an easy detectable landmark, and it has the advantage of not interfering with ongoing cardiopulmonary resuscitation (CPR), parallel IV insertion or assisted ventilation [3]. With FAST-R, the only option is sternal insertion. This site may not always be easily accessible, as it is close to the compression site during CPR. A blunt trauma to the sternum may also prevent FAST-placement [12]. This factor may explain why our physicians or flight paramedics may prefer EZ-IO for primary insertion.

Almost all medications and fluids, as well as blood components, can also be administered through a IO access [11]. Our findings support this approach, as our patients received necessary drugs, crystalloids, plasma or whole blood. In 3 patients, the IO remained unused. This finding may indicate that a certain overuse in this patient group will be inevitable.

We found a higher number of IO insertions during our study period compared to an earlier study in our service [3]. Increased use of IO may indicate that the threshold for using IO has decreased following increased user experience with IO in our service or that improved devices are available. All the insertions were made by trained physicians and paramedics with experience in establishing IV access. Compared to the study conducted by Sunde et al., we recorded a lower number of IO insertions in pediatric emergencies [3]. This difference may indicate that our crews have improved their skills in establishing IV access in younger patients. In our HEMS, IO is primarily used as a rescue technique if other attempts at vascular access fails, and the technique needs to be reliable. The use of IO is generally recommended as a rescue technique in critically ill or injured patients if intravenous access cannot be achieved. In situations in which the patients suffer from severe hypovolemic/hemorrhagic shock, IV access is difficult or impossible, and our operational, prehospital conditions make this issue even more challenging. Thus, research is essential to determine which device is the most efficient in a prehospital service [3]. Additional clinical studies comparing intraosseous devices and insertion sites in the prehospital environment are needed. New IO methods may require more research to determine the best IO device.

### Limitations of the study

A limitation in our study is the limited number of IO insertions, especially using the FAST-R method. Additionally, the evaluation on flow rate is based on the physician’s assessment, with no objective volume measurements. A randomized controlled study would be difficult to conduct, as FAST-R cannot be randomly inserted in all patients, excluding pediatric patients.

### Strengths

Our study has been performed prospectively in a prehospital setting, involving all the different factors one has to consider when establishing an intraosseous infusion in critically ill patients. The insertions were all made by the same HEMS crews with the same training and medical background, which we believe reduces the interpretation bias. This approach has given a picture of the functionality and efficiency of the method used, as well as complications that might not be reported in a controlled research environment.

## Conclusions

Intraosseous access has been shown to be a reliable rescue technique in our HEMS with high first-pass success rates. EZ-IO seems to be a more rapid method to gain vascular access compared to FAST-R. However, FAST-R might be a better choice when high-flow infusions in adults are indicated. Few complications were registered with both devices.

## Abbreviations

CPR: Cardiopulmonary resuscitation
EZ-IO: Arrow EZ-IO Vascular Access System
FAST-R: FAST responder
HEMS: Helicopter emergency medical service
IO: Intraosseous
IV: Intravenous
